# Supplementary material for: β-Cyclodextrin Inhibits Monocytic Adhesion to Endothelial Cells through Nitric Oxide-Mediated Depletion of Cell Adhesion Molecules
Source: Molecules. 2020 Aug 6;25(16):3575. doi: 10.3390/molecules25163575 (PMC7464935; doi:10.3390/molecules25163575)
Supplement: Supplementary file 1 [file molecules-25-03575-s001.pptx]

## Slide 1
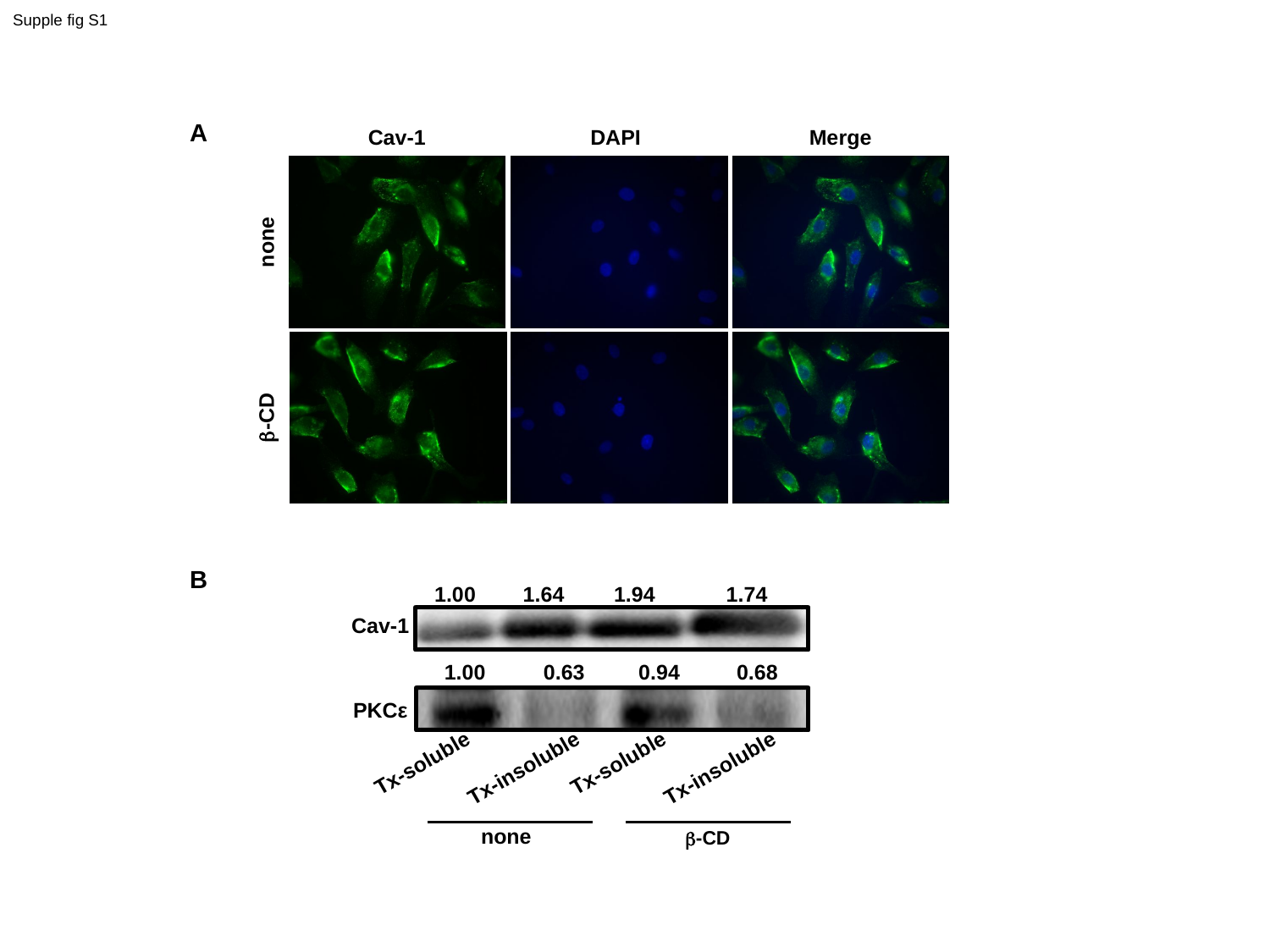

Supple fig S1
A
Cav-1
DAPI
Merge
none
b-CD
B
1.00
1.64
1.94
1.74
1.00
0.63
0.94
0.68
Tx-soluble
Tx-soluble
Tx-insoluble
Tx-insoluble
none
b-CD
Cav-1
PKCε

## Slide 2
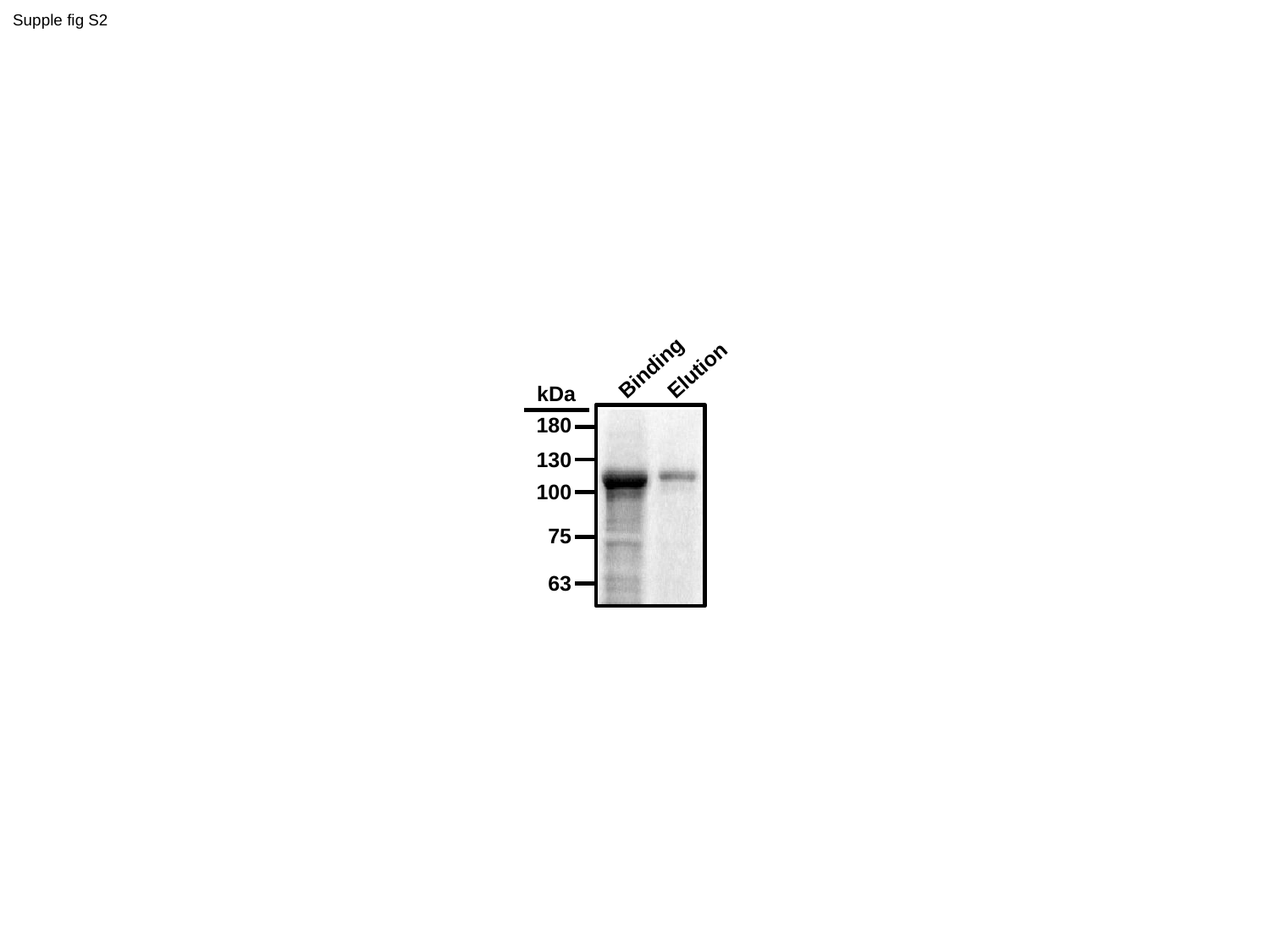

Supple fig S2
Binding
Elution
kDa
180
130
100
75
63

## Slide 3
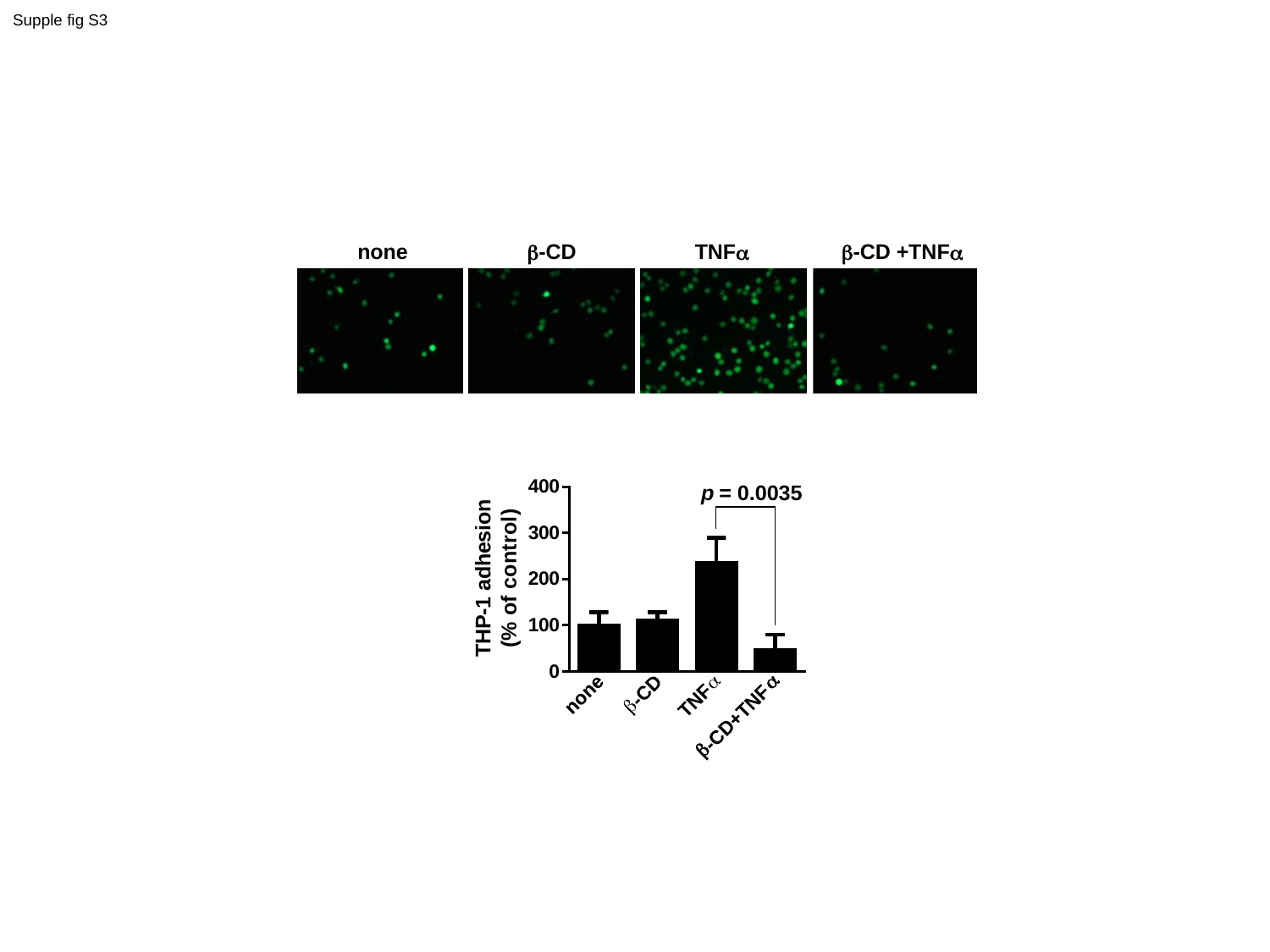

Supple fig S3
none
b-CD
TNFa
b-CD +TNFa

## Slide 4
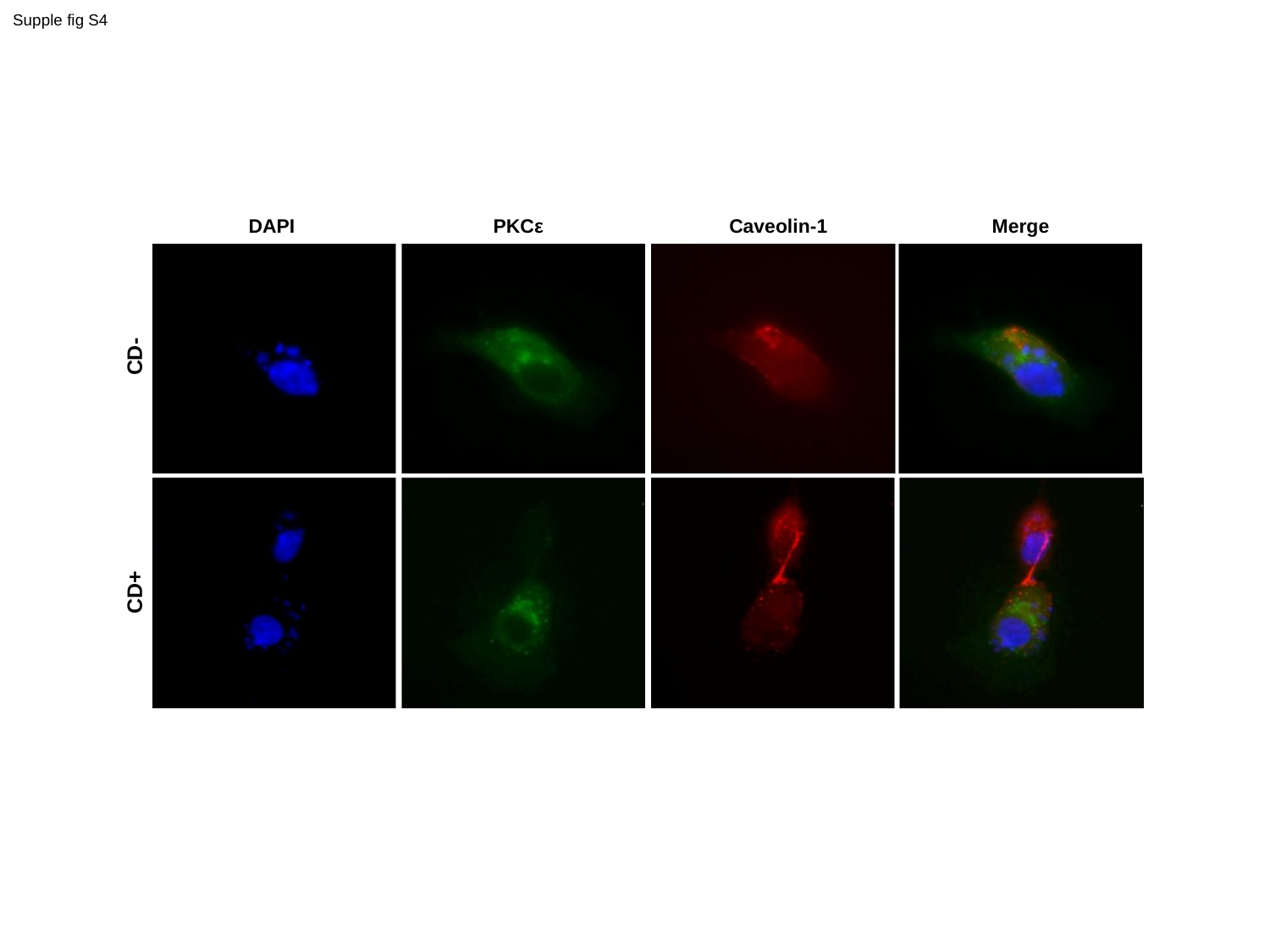

Supple fig S4
DAPI
PKCε
Caveolin-1
Merge
CD-
CD+
